# Perinatal ischemic stroke in an infant with factor VII deficiency: A CARE-compliant case report

**DOI:** 10.1097/MD.0000000000043710

**Published:** 2025-08-01

**Authors:** I-Ling Chou, Ting Wei Su, William James Lew, Hana F. Azizi, Xiaohua Zhou, Pei-Yu Yang

**Affiliations:** a School of Medicine, China Medical University, Taichung, Taiwan; b John A. Burns School of Medicine, Honolulu, HI; c Department of Rehabilitation and Regenerative Medicine, Columbia University Medical Center, New York, NY; d Department of Physical Medicine & Rehabilitation, Heersink School of Medicine, University of Alabama at Birmingham, Birmingham, AL; e Department of Physical Medicine and Rehabilitation, China Medical University Hospital, Taichung, Taiwan.

**Keywords:** case report, congenital factor VII deficiency, neonatal arterial ischemic stroke, perinatal stroke, rehabilitation

## Abstract

**Rationale:**

Hemophilia, an inherited bleeding disorder, is rarely caused by congenital factor VII (FVII) deficiency, occurring in 1 in 500,000 patients. Oftentimes, the presenting symptom of newborns with congenital FVII deficiency is intracranial hemorrhage, which can lead to adverse neurological outcomes.

**Patient concerns:**

A 2-day-old male infant, born late preterm, presented with symptoms of coffee-ground emesis, lethargy, and feeding difficulties.

**Diagnoses:**

Computed tomography revealed acute ischemic stroke with hemorrhagic transformation in the territory of the left middle cerebral artery, along with acute subdural hematoma and intraventricular hemorrhage. A few days later, magnetic resonance imaging showed an additional hemorrhagic infarction in the left parietal-occipital lobes. Hematologic testing confirmed FVII deficiency.

**Interventions:**

The patient underwent treatment with recombinant FVII. On the 15^th^ day, following the correction of the bleeding tendency, a ventriculoperitoneal shunt was inserted. An early and comprehensive rehabilitation program was initiated.

**Outcomes:**

The patient exhibits right-sided hemiplegia, visual deficits, and global developmental delays. However, gradual progress has been noted through rehabilitative interventions. Currently, at the age of 44 months, the patient can stand with minimal assistance for 10 minutes, cruise over extended distances, articulate brief phrases, and comply with simple instructions.

**Lessons:**

This case report describes the first documented neonatal case of congenital FVII deficiency associated with simultaneous cerebral infarction and hemorrhage, indicating that FVII deficiency does not protect against thrombosis. Early, comprehensive rehabilitation capitalizes on neuroplasticity, potentially enhancing motor, cognitive, and communicative skills and supporting functional independence in patients with perinatal stroke.

## 1. Introduction

Congenital factor VII (FVII) deficiency is a rare coagulopathy that often leads to intracranial hemorrhage (ICH) in neonates and poses considerable neurological risks. In this case report, we describe the first documented neonatal case of congenital FVII deficiency associated with simultaneous cerebral infarction and hemorrhage. We also reviewed congenital FVII deficiency and its relationship with thromboembolic events, neonatal arterial ischemic stroke (NAIS), and rehabilitation strategies for NAIS.

## 2. Case report

**Figure 1.**
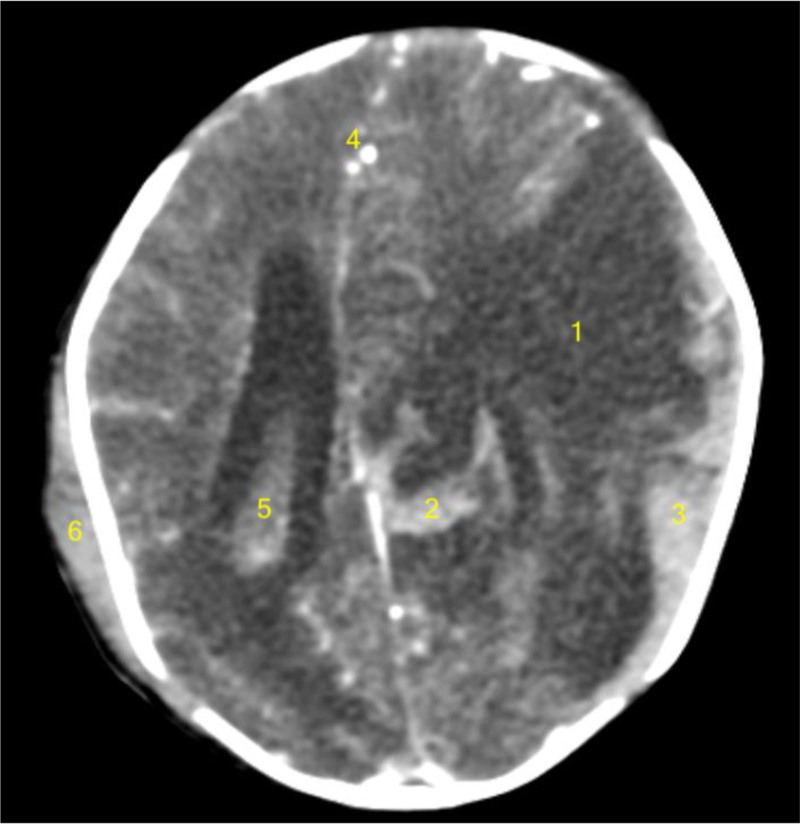
Brain CT at 2-day-old, axial section, brain window. A large area of acute brain tissue edema mixed with hemorrhage in the left frontal-temporal-parietal lobes and basal ganglion, acute ischemic stroke (1) with hemorrhagic transformation (2) of the left MCA territory. There was also a large acute subdural hematoma (3) in the left frontal-temporal-parietal region. This causes a strong mass effect, compressing the left lateral ventricle and shifting the midline to the right side (4). There was also intraventricular hemorrhage (IVH) in all the ventricles (5) with associated hydrocephalus, and severe subcutaneous swelling with hemorrhage (6) in the right frontal-parietal region. CT = computed tomography, IVH = intraventricular hemorrhage.

A two-day-old male infant, born late preterm at 36 + 4 weeks, was admitted with coffee-ground vomiting, lethargy, and feeding difficulties. Neurological assessment indicated a reduced light reflex, measuring 3.0 mm bilaterally, and decreased activity levels. The patient, weighing 2775 g at birth, was delivered via cesarean section to healthy, non-consanguineous parents. He had no known family history of hematological, neurological, vascular, metabolic, or immune diseases. Initial brain computed tomography revealed an acute ischemic stroke with hemorrhagic transformation in the left middle cerebral artery (MCA) territory, an acute subdural hematoma in the left frontal-temporal-parietal region, and intraventricular hemorrhage with hydrocephalus (Fig. 1). As part of the stroke risk evaluation, an echocardiogram was performed, revealing a patent foramen ovale (0.3–0.4 cm) with left-to-right shunting and trivial tricuspid regurgitation. No structural heart defects or vegetations were observed. The findings were deemed noncontributory to the patient’s clinical presentation. Hematological evaluation showed prolonged a prothrombin time (PT) of 53.4 seconds and normal levels of factors VIII and IX. A broad coagulopathy study was conducted.

**Figure 2.**
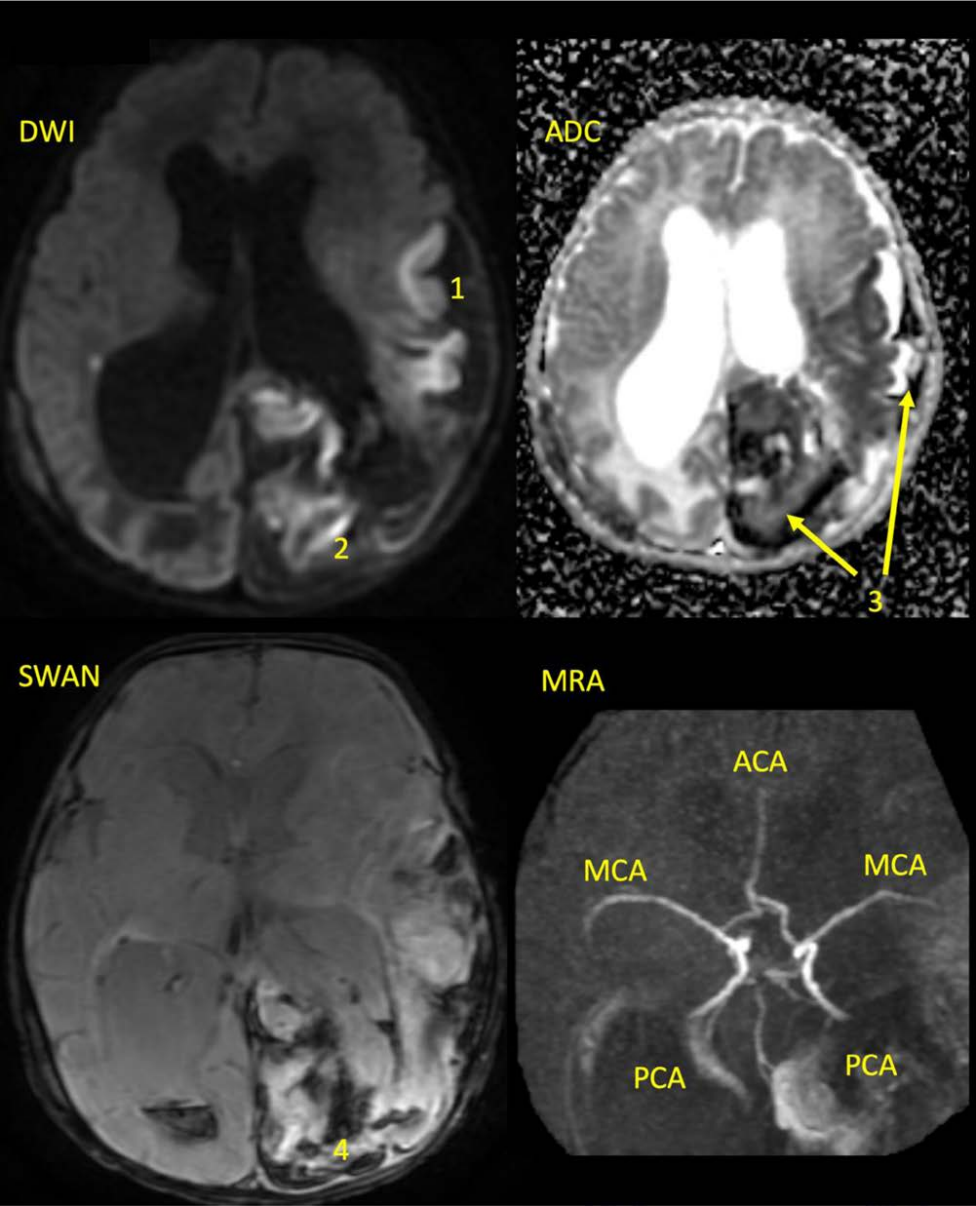
Brain MRI at 6-day-old. DWI: a large area of brain tissue in the left frontal-temporo-parietal lobe (1) and occipital lobe (2) is bright (hyperintense), indicating diffusion restriction. This finding suggested cytotoxic edema due to acute ischemic stroke of the left MCA and PCA. ADC map: the infarction area is dark (hypo intensity) (3) because of the low diffusion coefficient. SWAN: hemorrhage (4) due to acute ischemic stroke of the left PCA territory with hemorrhagic transformation. MRA: no occlusion of left MCA or PCA. ACA = anterior cerebral artery; ADC = apparent diffusion coefficient; DWI = diffusion weighted imaging; MRA = magnetic resonance angiography; MRI = magnetic resonance imaging; PCA = posterior cerebral artery; SWAN = susceptibility-weighted angiography.

Antibiotics, vitamin K, fresh frozen plasma, and cryoprecipitate were administered. Neurosurgery recommended the option of decompression craniectomy in accordance with clinical guidelines. Given the patient’s bleeding diathesis, the family was informed of the risks of intraoperative and postoperative hemorrhage. Upon understanding the potential complications, they made an informed decision to defer craniectomy. Despite treatment, the patient’s mental status deteriorated. He exhibited decreased activity, reduced mobility in his right extremities, and dysphagia. An orogastric tube was inserted due to the patient’s dysphagia. By day 4, the infant had experienced tonic-clonic seizures followed by a coma. Magnetic resonance imaging demonstrated an additional left posterior cerebral hemorrhagic infarction in the parietal-occipital lobes (Fig. 2). A subsequent coagulopathy workup, including assays for vWF activity, vWF antigen, FVII, factor VIII, factor IX, and fibrinogen, revealed a FVII level of <1%. FVII deficiency was diagnosed, and treatment with Eptacog Alfa (activated recombinant FVII) was initiated at a dose of 40 mcg/kg/day. Posttreatment, PT enhanced to 8.8 seconds, and FVII levels increased to 131.9%. Once the patient’s bleeding tendency was rectified, a ventriculo-peritoneal shunt was inserted on the 15th day, resolving the hydrocephalus. Despite this treatment, right hemiplegia with spasticity, dysphagia, and diminished visual and auditory responses persisted. An early intervention rehabilitation program was started to promote motor development, reduce spasticity, and improve swallowing function. An orthosis was also prescribed to prevent right ankle contracture. Following swallow training, the orogastric tube was removed on day 23, and the patient was discharged on day 33. He was adherent to regular FVII (Beriplex) injections (1 vial containing 500 IU administered every other day) and to his rehabilitation program, maintaining FVII activity levels above 70%. FVII levels were monitored every 3 months.

At 6 months, the infant exhibiting altered consciousness and reduced activity, was readmitted for ICH in the right frontal-parietal region. He received conservative treatment, including factor injections and supportive care. After a month, he was discharged with stable vital signs, clear consciousness, and improved activity. Further genetic testing detected a pathogenic mutation point, namely, F7:c.681 + 1G>T homozygous mutation. The patient’s clotting function is sustained at a level of 70% through factor injections, and no new instances of bleeding have occurred. A follow-up cranial ultrasound at 7 months demonstrated asymmetric ventriculomegaly, more pronounced on the left than the right, along with cystic encephalomalacia, findings consistent with a prior infarction and hemorrhagic insult.

The patient, who was suffering from spastic right hemiplegia, visual impairment, and global development delay, underwent a continuous rehabilitation program. His right limbs were at Brunnstrom stage III with a muscle power of 3/5 on the Medical Research Council scale. Modified Ashworth scales showed spasticity levels of one in the right upper limb and 2 in the right lower limb. A comprehensive rehabilitation program was initiated, including physical therapy to promote motor development and facilitate right limb movement, occupational therapy to improve upper limb function and reduce spasticity, along with vision, cognitive and speech therapy. Notable milestones included sitting and producing words at 1 year, and creeping at 20 months. Now, at the age of 44 months, his Bayley Scales of Infant Development (III) assessment showed cognitive skills at 10 months, receptive language at 14 months, and expressive language at 19 months. His fine motor and gross motor age corresponded to 10 months. He could stand with minimal assistance for 10 minutes, cruise over extended distances, articulate brief phrases, and comply with simple instructions. The patient’s standing balance has shown improvement following the administration of botulinum toxin injections. Based on the observations of the parents and clinical examinations, continued rehabilitation gradually enhances motor, speech, and cognitive functions.

The informed consent of the legal guardian was obtained for this case report. This study conforms to all CARE guidelines and reports the required information accordingly (see Supplementary Checklist, Supplemental Digital Content, https://links.lww.com/MD/P595).

## 3. Discussion

### 3.1. Congenital FVII deficiency

FVII is a plasma glycoprotein that initiates coagulation via tissue factors in the extrinsic pathway. The F7 gene, through 3 mRNA transcripts, encodes the FVII protein. Therefore, a mutation in the F7 gene disrupts FVII coding.^[1]^ FVII deficiency, defined as FVII levels below 50% of normal, affects approximately 1 in 500,000 individuals.^[2]^ The International Society on Thrombosis and Hemostasis classifies the severity of FVII deficiency as severe (<10%), moderate (10–20%), and mild (20–50%); bleeding risk is reliably indicated by clinical presentation.^[3]^ Severe deficiency is often concomitant with central nervous system hemorrhage, gastrointestinal bleeding, or hemarthrosis. Newborns are particularly vulnerable to ICH, which is usually associated with infant mortality.^[4]^ However, mild cases of ICH may remain asymptomatic. Typically, a diagnosis comprises a workup of a patient’s PT, PTT, aPTT, and platelet count and a measurement of FVII coagulation activity after the patient presents with a bleeding episode. However, the severity of FVII deficiency does not correlate with the PT or bleeding severity of the patient.^[5]^ Treatments are based on deficiency type and the patient’s economic and geographic factors. Our patient was treated with activated recombinant FVII, which is noted for its safety in long-term use due to its prolonged effects. Alternatives, such as plasma-derived FVII and prothrombin complex concentrates, have short half-lives, and fresh frozen plasma concentrates are considered as a last resort owing to their potential risks.^[6]^

### 3.2. Thromboembolic events and congenital FVII deficiency

Our patient exhibited severe FVII deficiency. Paradoxically, he presented with an acute ischemic stroke, where 3% to 4% of FVII deficiency cases involved thrombosis. In a cohort of 514 adult patients, 9 experienced thrombosis, mostly postoperative, with only 3 cases being spontaneous.^[7]^ Marty et al noted a range of thrombotic manifestations including deep vein thrombosis, superficial vein thrombosis, and pulmonary embolism, with young adults (aged 16–52 years) being more prone to atypical thrombotic sites.^[8]^ Girolami et al reported that thrombotic events can occur across all severities of FVII deficiency, particularly when compounded by additional risk factors, such as surgery and old age.^[9]^ The interplay between diverse risk factors complicates the assessment of individual contributions to thrombosis.

In pediatric cases, this thrombotic tendency is absent. In neonates, thrombotic events associated with FVII deficiency are exceedingly rare and remain poorly understood. A study reported that among 73 children with FVII deficiency, 17 exhibited severe bleeding, 8 had central nervous system bleeding, and none experienced thrombotic events.^[10]^ These findings suggest that thrombosis in FVII deficiency typically requires adult-related risk factors and FVII deficiency does not preclude thrombosis. The immature neonatal coagulation system may engage compensatory mechanisms, and factors such as perinatal hypoxia, endothelial injury, or paradoxical embolism (e.g., via a patent foramen ovale) may contribute to thrombotic risk. Although no prothrombotic genetic mutations were identified in our patient, the concurrent ischemic stroke may have resulted from unrecognized transient prothrombotic triggers during the perinatal period.

### 3.3. Neonatal arterial ischemic stroke

Perinatal stroke occurs from 20 weeks of gestation to 4 weeks postnatally and affects approximately 1 in 2000 live births.^[11]^ NAIS accounts for 80% to 90% of perinatal stroke and typically presents as seizures within 12 to 72 hours after delivery; it is diagnosed via magnetic resonance imaging showing ischemic infarction in arterial territories, primarily within the MCA.^[12]^

Its pathophysiological mechanism remains unknown, but hypotheses include placental thromboembolic events, cardiac diseases, bacterial meningitis, male sex, primiparity, and cesarean section.^[13]^ In our patient, minor factors such as a cesarean delivery were observed.

Our patient’s imaging results illustrated concurrent ischemic and hemorrhagic strokes, causing challenges in ascertaining the order of events. Hemorrhagic transformation following acute ischemic stroke occurs in 18% to 42% of cases and is often due to the disruption of the blood–brain barrier.^[14]^ Conversely, ischemic stroke occurring after a hemorrhagic event is uncommon, with potential explanations including the vasospasm of nearby vessels, increased intracranial pressure causing vascular compression, and clot formations at the site of hemorrhage.^[15]^

NAIS manifestations vary but primarily include seizures presenting in the form of unilateral, repetitive clonic seizures in otherwise healthy, full-term infants within 1 to 3 days after birth. The prognosis worsens if NAIS co-occurs with other stroke types, especially hemorrhage. Patients may exhibit apnea, encephalopathy, feeding issues, irritability, and fever.^[12]^

The acute management of NAIS and perinatal hemorrhagic stroke emphasizes supportive care from a multidisciplinary team.^[11]^ Seizures are commonly controlled by using phenobarbital for a minimal duration to mitigate neurotoxicity.^[16]^ For severe hemorrhage causing acute obstructive hydrocephalus or brainstem compression, surgical interventions such as craniectomy, hematoma evacuation, or external ventricular drainage may be necessary.^[17]^ Tailored hematological evaluations are crucial for addressing potential coagulopathies.

### 3.4. Early intervention and rehabilitation strategies for neonatal stroke

Perinatal stroke can lead to lasting conditions, such as hemiparetic cerebral palsy and epilepsy, yet subtle symptoms often delay diagnosis. In our case, coffee-ground vomiting, lethargy, and common indicators for stroke were lacking. Most infants with NAIS are diagnosed between 12 and 72 hours of age, underscoring the need for heightened awareness.^[11]^

Early recognition and intervention can mitigate neurological deficits, with rehabilitation as the primary treatment. Unlike adult stroke, neonatal stroke rehabilitation aids skill acquisition in the developing brain, where new functions are still emerging.^[18]^

Neonatal stroke can disrupt the development or acquisition of certain functions. To improve motor function, intensive manual training is suggested. The most compelling evidence of efficacy is shown by constraint-induced movement therapy and bimanual training. Constraint-induced movement therapy promotes the use of an impaired limb by limiting the less impaired one through casting or immobilization, whereas bimanual training focuses on incorporating the impaired limb into two-handed activities.^[18]^ Occupational therapy can improve sensory processing and sensorimotor integration, benefiting motor and cognitive skills. Injections of botulinum toxin type A can reduce spasticity and increase range of motion. Repetitive transcranial magnetic stimulation and transcranial direct current stimulation may also boost motor skills in hemiparetic children by modulating cortical excitability and neuronal plasticity. However, their exact mechanisms and effects need further study.^[18]^

Language, cognitive, and behavioral deficits constitute an important part of the impairments following neonatal stroke. These deficits may develop over time, potentially exacerbating in the long run.^[12]^ Commonly affected cognitive areas include attention, working memory, and executive functions. The 2 primary intervention strategies are substitution and restoration. Substitution adjusts the environment to offset deficits, whereas restoration uses standardized programs to train cognitive functions.^[19]^ Speech-language therapy primarily targets basic language skills (e.g., Naming) or motor speech function (e.g., dysarthria).

Most patients with neonatal stroke develop hemiplegic cerebral palsy. The ischemic and hemorrhagic strokes in our patient resulted in bilateral brain involvement, affecting not only motor, speech, and cognitive functions but also visual ability. Additionally, his bleeding diathesis limited the intensity of his rehabilitation program. The complexity of his condition contributed to his more limited progress compared with those of other patients with cerebral palsy resulting from perinatal stroke. However, with early intervention, his dysphagia improved rapidly, and his orogastric tube was removed by day 23. While his language development was delayed due to left MCA involvement, he began to produce words by the age of 1 year. His standing balance has shown improvement following the administration of botulinum toxin injections. Alongside medical management, he was also introduced to educational resources.

Owing to rapid nervous system development, early detection and targeted intervention can help reroute corticospinal fibers from the affected hemisphere, leveraging a critical neuroplasticity window to maintain motor pathway development.^[18]^

## 4. Conclusions

This case report indicates that FVII deficiency does not prevent thrombosis. Further research is needed due to the unclear risk factors and pathophysiology of ischemic stroke in patients with FVII deficiency.

Clinical presentation is crucial for treatment decisions in patients with perinatal stroke, highlighting the need for individualized care. Early, comprehensive rehabilitation leverages neuroplasticity to improve motor, cognitive, and communicative skills and promote functional independence in patients with perinatal stroke.

## Author contributions

**Conceptualization:** Pei-Yu Yang.

**Data curation:** I-Ling Chou, Ting Wei Su, William James Lew.

**Formal analysis:** I-Ling Chou, Ting Wei Su, William James Lew.

**Project administration:** Pei-Yu Yang.

**Supervision:** Pei-Yu Yang.

**Writing – original draft:** I-Ling Chou, Ting Wei Su.

**Writing – review & editing:** Hana F. Azizi, Xiaohua Zhou, Pei-Yu Yang.

## Supplementary Material


